# Implementation of an e-Learning course in physical activity and sedentary behavior for pre- and in-service early childhood educators: Evaluation of the TEACH pilot study

**DOI:** 10.1186/s40814-022-01015-1

**Published:** 2022-03-17

**Authors:** Brianne A. Bruijns, Leigh M. Vanderloo, Andrew M. Johnson, Kristi B. Adamo, Shauna M. Burke, Valerie Carson, Rachel Heydon, Jennifer D. Irwin, Patti-Jean Naylor, Brian W. Timmons, Patricia Tucker

**Affiliations:** 1 Health and Rehabilitation Sciences Program, Faculty of Health Sciences, Western University, London, ON Canada; 2 ParticipACTION, Toronto, ON Canada; 3 School of Occupational Therapy, Faculty of Health Sciences, Western University, 1201 Western Road, Elborn College, Room 2547, London, ON N6G 1H1 Canada; 4 School of Health Studies, Faculty of Health Sciences, Western University, London, ON Canada; 5 School of Human Kinetics, Faculty of Health Sciences, University of Ottawa, Ottawa, ON Canada; 6 Children’s Health Research Institute, London, ON Canada; 7 Faculty of Kinesiology, Sport, and Recreation, University of Alberta, Edmonton, AB Canada; 8 Faculty of Education, Western University, London, ON Canada; 9 School of Exercise Science, Physical and Health Education, University of Victoria, Victoria, BC Canada; 10 Child Health and Exercise Medicine Program, McMaster University, Hamilton, ON Canada

**Keywords:** e-Learning, Physical activity, Sedentary behavior, Early childhood educators, Childcare, Implementation, Process evaluation

## Abstract

**Background:**

Childcare-based physical activity (PA) and sedentary behavior (SB) interventions have traditionally used in-person training to supplement early childhood educators’ (ECEs) knowledge and confidence to facilitate physically active programming for the children in their care. However, this method of delivery is resource-intensive and unable to reach a high number of ECEs. The purpose of the Training pre-service EArly CHildhood educators in PA (TEACH) pilot study was to test the implementation (e.g., fidelity, feasibility, acceptability) of an e-Learning course targeting PA and SB among a sample of pre-service (i.e., post-secondary students) and in-service (i.e., practicing) ECEs in Canada.

**Methods:**

A pre-/post-study design was adopted for this pilot study, and implementation outcomes were assessed cross-sectionally at post-intervention. Pre-service ECEs were purposefully recruited from three Canadian colleges and in-service ECEs were recruited via social media. Upon completing the e-Learning course, process evaluation surveys (*n* = 32 pre-service and 121 in-service ECEs) and interviews (*n* = 3 pre-service and 8 in-service ECEs) were completed to gather ECEs’ perspectives on the e-Learning course. Fidelity was measured via e-Learning course metrics retrieved from the web platform. Descriptive statistics were calculated for quantitative data, and thematic analysis was conducted to analyze qualitative data.

**Results:**

Moderate-to-high fidelity to the TEACH study e-Learning course was exhibited by pre-service (68%) and in-service (63%) ECEs. Participants reported that the course was highly acceptable, compatible, effective, feasible, and appropriate in complexity; however, some ECEs experienced technical difficulties with the e-Learning platform and noted a longer than anticipated course duration. The most enjoyed content for pre- and in-service ECEs focused on outdoor play (87.5% and 91.7%, respectively) and risky play (84.4% and 88.4%, respectively).

**Conclusions:**

These findings demonstrate the value of e-Learning for professional development interventions for ECEs. Participant feedback will be used to make improvements to the TEACH e-Learning course to improve scalability of this training.

**Supplementary Information:**

The online version contains supplementary material available at 10.1186/s40814-022-01015-1.

## Key messages regarding feasibility

- With the recent shift to virtual platforms for professional learning interventions for early childhood educators (ECEs), little is known about the feasibility of using e-Learning to deliver physical activity and sedentary behavior-related training among this population.
- This pilot study showed that the TEACH study e-Learning course was well-received by both pre-service and in-service ECEs and that it improved their knowledge and confidence to facilitate more physically active and less sedentary programming. Both groups also reported that the e-Learning platform was convenient to work into their schedules, promoted their learning, and would be feasible to integrate into pre- and in-service ECE training.
- Findings from this study will be used to make improvements to the e-Learning course (e.g., enhancing mobile compatibility, creating shorter modules) to promote scalability of the intervention.

## Background

Early childhood educators (ECEs) are important role models for young children (<5 years) in childcare [1] and can profoundly influence their movement behaviors (e.g., physical activity, sedentary behavior [i.e., exerting little energy in a sitting/reclining posture]) [2]. In fact, ECEs’ confidence [3] and values [4] regarding physical activity, as well as their own physical activity levels [2, 5] and the amount of physical activity-related training they have completed [6, 7], have all been associated with children’s physical activity levels in childcare. Given the importance of promoting healthy movement behaviors in early childhood [8], which is when young children establish health-related habits [9], it is essential that ECEs are educated about physical activity and sedentary behavior and engaged in health-promoting practices themselves so that they are confident, willing, and able to incorporate appropriate amounts of high-quality movement experiences for children in their care.

Although sedentary behavior-related content is largely missing from existing professional learning initiatives, several previous childcare-based interventions have included physical activity training for ECEs [10–17]; many of which have been successful at increasing young children’s physical activity while in care [11–13, 16]. For example, an intervention led by Pate and colleagues (2016), involving in-person training for ECEs regarding the promotion of structured and unstructured physical activity and active learning, was shown to be effective at increasing preschoolers’ (*n* = 379) moderate-to-vigorous physical activity (MVPA) [12]. Similarly, Hoffman and colleagues (2020) administered online training in physical activity for ECEs, and children whose educators received the training increased their daily MVPA by nearly 13 min [13]. However, mixed results have been noted regarding the effectiveness of training interventions at improving ECEs’ knowledge and confidence regarding physical activity; some studies have reported improvements in these outcomes [3, 18], while others have reported no change [19]. While measuring effectiveness of interventions is important, it is beneficial to look at implementation outcomes and determinants of both effective and ineffective interventions to provide context as to which components of implementation help or hinder intervention success.

To guide researchers regarding the implementation and scale-up of interventions relating to physical activity and nutrition, McKay and colleagues [20] conducted a Delphi study to generate consensus on implementation and scale-up frameworks, indicators, and measures. From this study [20], a minimum set of implementation outcomes (*n* = 5) and determinants (*n* = 10) was created, which included indicators such as fidelity, sustainability, acceptability, and feasibility (among others). Previous childcare-based ECE training interventions have reported on these implementation outcomes and determinants; frequently, fidelity and acceptability scored high [21–23], while mixed results have been found for feasibility [21, 22]. These findings provide insight into which implementation outcomes and determinants (e.g., feasibility) should be targeted with greater attention and support in future ECE physical activity training interventions to achieve better success.

While a number of childcare-based physical activity interventions have included ECE training [10–17, 23], few have employed training as the sole intervention component [12–14, 16], and training was often used to educate ECEs about a physical activity-promoting program they were required to administer [12, 16, 17, 23] rather than to provide ECEs with general knowledge and strategies to facilitate active childcare settings. Additionally, a lack of focus in previous training interventions has been apparent concerning educating ECEs about sedentary behavior and risky play. Most studies only focus on physical activity uniquely [10, 12, 15, 17, 23] or in combination with nutrition education [18, 24]. However, with in-person training often reported as resource-intensive and lacking scalability, advances in training interventions for ECEs have since moved training online, via webinars and e-Learning courses [18, 19, 25, 26]. As such, the goal of the Training pre-service EArly CHildhood educators in physical activity (TEACH) study was to improve ECEs’ knowledge, confidence, and intentions regarding promoting healthy movement behaviors by providing comprehensive training in physical activity and sedentary behavior in childcare settings via an e-Learning course. To achieve this goal, a pilot study was undertaken to test the short-term efficacy and explore implementation of the e-Learning course with both pre-service ECEs (i.e., post-secondary students enrolled in an ECE program) and in-service ECEs (i.e., those who have completed their schooling and are employed in a childcare setting). This paper presents the evaluation undertaken to examine implementation of the TEACH pilot study.

## Methods

A pre-post (within-subjects) study design was employed for the TEACH pilot study, and implementation outcomes were measured cross-sectionally post-intervention via an online survey, interviews, and e-Learning course metrics. This process evaluation examined 13 implementation outcomes and determinants selected from recommendations by McKay et al. [20] and the *Consolidated Framework for Implementation Research* (CFIR) [27] and with consideration to those that were able to be measured within the pilot study design. These outcomes and determinants included dose delivered, fidelity, acceptability, feasibility, compatibility, complexity, self-efficacy, context, perceived effectiveness, perceived benefits, motivation, tension for change, and relative priority. See Table 1 for the TEACH pilot study implementation outcomes/determinants and the corresponding data source(s) and analyses. This study was approved by the Non-Medical Research Ethics Board at Western University (REB# 116816).

**Table 1:** Implementation outcomes and determinants of the TEACH pilot study

Implementation outcome/determinant	Question	Measurement tool/procedure	Data analyses
Dose delivered	To what degree were e-learning course modules completed?	e-Learning platform metrics	Module completion %
Fidelity (adherence)	What proportion of participants successfully completed the e-learning course?	e-Learning platform metrics	% of registered participants who successfully completed the e-Learning course
Acceptability	How satisfied were participants with the e-learning course?	Process evaluation survey; interviews	Descriptive statistics; thematic analysis
Feasibility	To what extent was the e-learning course easy and convenient to complete?	e-Learning metrics; interviews	# of days to complete the course; thematic analysis
Compatibility (appropriateness)	To what extent does the e-learning course fit with the mission, priorities, and values of the ECE profession?	Process evaluation survey; interviews	Descriptive statistics; thematic analysis
Complexity	To what extent was the e-Learning course difficult or easy to complete?	e-Learning platform metrics; process evaluation survey; interviews	*M* score across all module knowledge assessments; descriptive statistics; thematic analysis
Self-efficacy	How did participants perceive their ability to achieve e-learning course outcomes?	Process evaluation survey	Descriptive statistics
Context	What were the barriers and facilitators for completing the course?	Process evaluation survey; interviews	Descriptive statistics; thematic analysis
Perceived effectiveness	To what extent did the e-learning course increase participants’ knowledge about physical activity and sedentary behaviour? To what extent did the e-learning course design/method of delivery help them achieve learning outcomes?	Process evaluation survey; interviews	Descriptive statistics; thematic analysis
Perceived benefits	To what degree did participants feel the e-learning course was advantageous for their professional development?	Process evaluation survey; interviews	Descriptive statistics; thematic analysis
Motivation	What motivated participants to complete the course? To what extent did completing the course influence their interest in the topic?	Process evaluation survey; interviews	Descriptive statistics; thematic analysis
Tension for change	To what degree did participants feel current ECE educational opportunities in physical activity and sedentary behavior were lacking?	Interviews	Thematic analysis
Relative priority	To what extent did participants feel the e-learning course was important for those in their profession?	Process evaluation survey; interviews	Descriptive statistics; thematic analysis

*Note:* Implementation outcomes and determinants derived from McKay et al. (2019) and the *Consolidated Framework for Implementation Research* (Damschroder et al., 2009); *M* mean

### Study procedures and participant recruitment

Pre-service ECEs from three purposefully selected (based on location and class size) Canadian colleges with an early childhood education program were recruited; one college from Ontario, Alberta, and the Northwest Territories. In-service ECEs employed in various childcare settings across Canada were also recruited, via social media advertisements, to participate in this study. Participants were recruited from March to May 2021, and implied consent was given by commencing the first survey. For additional details about pilot study participants and recruitment, consult Bruijns et al. [28].

Following a baseline survey, pre- and in-service ECEs completed an e-Learning course in physical activity and sedentary behavior in early childhood. The course content was developed via a Delphi process [29], and the e-Learning course comprised four modules (each of which was approximately 90 min in length). To pass each module, participants needed to score 10 out of 12 correct responses on a knowledge assessment (which included multiple-choice and matching questions to test learners on module content). Unlimited attempts were provided to pass each assessment. Participants were encouraged to complete the e-Learning course within a 2-week timeframe; however, e-Learning accounts were not deactivated until the study closure date (i.e., participants were allowed to take more than 2 weeks to complete the course). Upon receiving their e-Learning course certificate, the participants were directed to a follow-up survey. Pre-service ECEs were required by their instructors to complete the e-Learning course in its entirety, but pre- and post-course surveys were completed voluntarily. One college provided in-person class time to complete the e-Learning course, while the other two colleges provided virtual (unmonitored) class time. In-service ECEs completed all study elements (i.e., surveys and the e-Learning course) on their own volition. For more details about the course and its development, consult the study protocol for the TEACH study (Tucker et al.: Training pre-service EArly CHildhood educators in physical activity (TEACH): Protocol for a quasi-experimental study, revision requested).

### Tools

#### e-Learning course metrics

Course metrics available through the web-based learning management system (LMS; i.e., *TalentLMS)* platform were retrieved, including the percent of registered learners who successfully completed the course (fidelity); completion rate of modules (dose delivered); percent of learners who passed each end-of-module knowledge assessment on the first, second, or third (or more) attempt (complexity); and the average number of days it took learners to complete the course (feasibility).

#### Process evaluation survey

An online process evaluation survey was developed and administered via Qualtrics for the purposes of this study, informed by the *Evaluating E-Learning System Success* (EESS) model [30]. The survey comprised 38 items, with 34 of these items rated on a 5-point Likert scale (1 = strongly disagree to 5 = strongly agree). These 34 items (Cronbach’s *α* = 0.98 and 0.94 for pre- and in-service ECEs, respectively) were grouped into the following implementation outcomes and determinants: *acceptability* (*n* = 10 items), *complexity* (*n* = 5 items), *self-efficacy* (*n* = 2 items), *compatibility* (*n* = 1 item), *perceived effectiveness* (*n* = 8 items), *perceived benefits* (*n* = 3 items), *content novelty* (*n* = 1 item), and *motivation* (*n* = 4 items). An additional four questions were designed to gather participants’ perspectives on the course content, delivery, challenges experienced, and suggestions for improvement (two of which allowed for open-ended responses).

#### Interviews

At the end of the follow-up survey, ECEs were asked whether they would participate in a 20 to 30-min Zoom interview to discuss their experiences with the course. Randomly selected volunteers from the pre- and in-service ECE study populations were contacted via email to schedule an interview time. Following verbal consent, all interviews were conducted by BAB using a semi-structured interview guide (Additional File 1) that was informed by codebook guidelines from the CFIR [31]. In the interviews, ECEs were asked to share their perspectives regarding their likes and dislikes about the course, the complexity of the course content and assessments, course elements that supported/hindered their learning, course content that was new to them, how the course compared to previous e-Learning courses they had taken, suggestions for improvement, and the extent to which they thought the course would integrate well into post-secondary early childhood education curricula. Saturation was reached after six interviews for in-service ECEs; however, two additional interviews were completed to confirm findings. Due to the small number of pre-service ECE volunteers, only three interviews were conducted. All interviews took place between April and May 2021 and were recorded and transcribed verbatim.

#### Data analysis

Descriptive statistics were conducted in Excel Workbook to analyze e-Learning course metrics and in SPSS (version 27) to analyze quantitative data from the online survey (independently by study group). Means (*M*) and standard deviations (*SD*) were calculated for average days needed to complete the course and Likert scale responses from the process evaluation survey. Frequencies were calculated to report the percent of learners who passed the course (in its entirety), total modules completed, learners who passed end-of-module knowledge assessments on the first attempt or multiple attempts, learners’ preferred/novel topic areas of the course, and course delivery elements (e.g., text, audio, video) that best supported participants’ learning. Using deductive pre-planned codes from the interview guide, thematic analysis was completed in QSR NVivo (version 12) to analyze interview transcripts and open-ended survey questions. Two researchers coded the interview transcripts independently and identified common themes within each study population (pre- and in-service ECEs). To minimize confirmation bias, a research assistant was recruited solely to code the data (and was not directly involved in the research project). Trustworthiness of the data was ensured throughout by following Patton’s [32] recommendations regarding credibility, confirmability, dependability, and transferability (e.g., member-checking).

## Results

### Participant demographics and e-learning course metrics

A total of 51 pre-service and 274 in-service ECEs were recruited for the pilot study. Of the 71^1^ and 199 pre- and in-service ECEs who registered for the course, 48 (67.6%) and 125 (62.8%) pre- and in-service ECEs successfully completed the course, respectively. For dose delivered, 93.9% and 90.5% of modules were completed by pre- and in-service ECEs, respectively. Across the four end-of-module knowledge assessments, 29.4% and 53.8% of pre- and in-service ECEs passed on the first attempt, 33.3% and 24.8% passed on the second attempt, and 37.3% and 21.4% needed three or more attempts to pass, respectively. The mean number of days it took pre- and in-service ECEs to complete the course was 4.3 (*SD* = 11.5) and 13.1 (*SD* = 12.3) days, respectively.

A total of 32 pre-service ECEs and 121 in-service ECEs completed the process evaluation survey (response rates of 62.7% and 44.2%, respectively). Pre-service ECEs were 26.7 years old (*SD* = 6.9), and the majority were female (93.8%). The most prevalent self-reported racial or cultural identities were South Asian (28.1%) or First Nations/Inuit/Métis (28.1%). Most participants reported having previous experience with e-learning courses/workshops (65.6%). In-service ECEs were 37.1 years old (SD = 9.5), and most were Caucasian (66.1%) and had experience with e-learning courses or workshops (70.2%). See Bruijns et al. (2022) [28] for complete participant demographics.

### Perspectives on course content and delivery

Pre-service ECEs reported enjoying the *Introduction to Physical Activity* (87.5%) and *Outdoor Play* (87.5%) topics the most and least enjoyed the content on *Creating Physical Activity and Sedentary Behaviour Policies* (15.6%). In-service ECEs enjoyed the content on *Loose Parts Play* the most (92.6%) and the *Video Library of Activities* the least (26.4%). For pre- and in-service ECEs, the top content areas that represented new topics for them were *How to Track and Set Goals for Movement Behaviours in Childcare* (37.5%) and *The Canadian 24-Hour Movement Guidelines for the Early Years* (46.3%), respectively. See Table 2 for frequencies of ECEs’ preferences and perspectives of novelty for all course topics.

**Table 2:** Pre- and in-service early childhood educators’ preference for and novelty of topic areas in the e-learning course

Topic	Enjoyed topic the most ***N*** (%)		Enjoyed topic the least ***N*** (%)		Topic was new to them ***N*** (%)	
	Pre-service (***N*** = 32)	In-service (***N*** = 121)	Pre-service (***N*** = 32)	In-service (***N*** = 121)	Pre-service (***N*** = 32)	In-service (***N*** = 121)
Introduction to physical activity	28 (87.5)	99 (81.8)	2 (6.3)	14 (11.6)	4 (12.5)	5 (4.1)
Introduction to sedentary behavior	21 (65.6)	85 (70.2)	4 (12.5)	14 (11.6)	8 (25.0)	22 (18.2)
The Canadian 24-Hour Movement Guidelines for the Early Years	20 (62.5)	69 (57.0)	4 (12.5)	7 (5.8)	9 (28.1)	56 (46.3)
Physical literacy	27 (84.4)	93 (76.9)	0 (0.0)	4 (3.3)	6 (18.8)	25 (20.7)
Fundamental movement skills	24 (75.0)	96 (79.3)	2 (6.3)	9 (7.4)	6 (18.8)	18 (14.9)
Factors that influence physical activity and sedentary behavior in childcare	22 (68.8)	94 (77.7)	4 (12.5)	6 (5.0)	8 (25.0)	21 (17.4)
Outdoor play	28 (87.5)	111 (91.7)	1 (3.1)	3 (2.5)	1 (3.1)	1 (.8)
Risky play	27 (84.4)	107 (88.4)	1 (3.1)	2 (1.7)	3 (9.4)	18 (14.9)
Loose parts play	23 (71.9)	112 (92.6)	2 (6.3)	4 (3.3)	7 (21.9)	11 (9.1)
How to track and set goals for movement behaviors in childcare	18 (56.3)	58 (47.9)	3 (9.4)	22 (18.2)	12 (37.5)	46 (38.0)
Role modelling appropriate movement behaviors	25 (78.1)	102 (84.3)	3 (9.4)	10 (8.3)	2 (6.3)	7 (5.8)
How to modify your teaching behaviors to support activity	26 (81.3)	96 (79.3)	2 (6.3)	7 (5.8)	5 (15.6)	15 (12.4)
Programming physical activity	24 (75.0)	100 (82.6)	2 (6.3)	5 (4.1)	8 (25.0)	15 (12.4)
Programming active breaks, transitions, and learning opportunities to minimize sedentary behavior	26 (81.3)	92 (76.0)	1 (3.1)	4 (3.3)	10 (31.3)	25 (20.7)
Getting families on board	24 (75.0)	80 (66.1)	2 (6.3)	17 (14.0)	7 (21.9)	24 (19.8)
Creating physical activity and sedentary behavior policies	19 (59.4)	63 (52.1)	5 (15.6)	21 (17.4)	11 (34.4)	46 (38.0)
Professional learning opportunities	23 (71.9)	46 (74.4)	3 (9.4)	9 (7.4)	7 (21.9)	31 (25.6)
Resources for early childhood educators	24 (75.0)	87 (71.9)	2 (6.3)	10 (8.3)	7 (21.9)	34 (28.1)
Video library of activities	21 (65.6)	69 (57.0)	4 (12.5)	32 (26.4)	8 (25.0)	24 (19.8)

*Note:* Participants were directed to “check all that apply” when selecting their most/least preferred topics and topics that were new to them

**Table 3:** Pre- and in-service early childhood educators’ perspectives on e-learning course implementation

Item	Pre-service (***N*** = 32)		In-service (***N*** = 121)	
	***M***	***SD***	***M***	***SD***
***Acceptability***				
Overall, I enjoyed using the course	4.55	.81	4.69	.78
Overall, I was satisfied with the course	4.61	.803	4.69	.70
The course provided me with sufficient information about *physical activity* in early childhood	4.69	.69	4.80	.42
The course provided me with sufficient information about *sedentary behavior* in early childhood	4.71	.69	4.72	.50
The course met my requirements	4.58	.81	4.74	.54
The design of the course (e.g., fonts, style, colours, images, videos) was acceptable	4.52	.81	4.74	.46
The course used interesting and appropriate delivery methods (e.g., animation, video, audio, text, simulation, etc.)	4.65	.80	4.50	.73
The evaluation and assessment components of the e-Learning course were appropriate based on course content presented	4.55	.93	4.56	.69
I had enough time to complete the course	4.71	.69	4.60	.71
The length of each module within the e-Learning course was appropriate	4.52	.93	4.31	1.04
***Complexity***				
It was easy to use the course	4.68	.60	4.61	.76
The course was flexible to navigate	4.61	.76	4.47	.90
There were clear instructions about how to use the course	4.71	.59	4.74	.59
The structure of the course was well organized into understandable components	4.68	.79	4.79	.58
Information presented in the course was concise and clear	4.65	.80	4.74	.66
***Self-efficacy***				
My previous experience with e-learning systems and/or computer applications helped me in using the course	4.65	.76	4.16	.90
I was able to perform tasks in the course successfully	4.68	.70	4.68	.57
***Compatibility***				
Taking the course was a useful experience to complement my early childhood education training	4.71	.78	4.64	.76
***Perceived effectiveness***				
The course helped me learn effectively	4.63	.85	4.55	.84
The course was an effective educational tool	4.73	.79	4.74	.66
The course helped me to achieve the learning outcomes of each module	4.55	.89	4.65	.72
The course increased my knowledge about *physical activity* in early childhood	4.56	1.10	4.64	.69
The course increased my knowledge about *sedentary behavior* in early childhood	4.62	.94	4.50	.91
The within-module knowledge checks helped facilitate my learning	4.58	.81	4.52	.74
The end-of-module knowledge assessments helped facilitate my learning	4.58	.85	4.45	.85
The e-learning mode of delivery helped me learn as effectively as in-person instruction	4.42	1.06	4.48	.81
***Perceived benefits***				
The knowledge I gained from this course will be useful to me as an early childhood educator	4.74	.77	4.79	.62
Access to this course would be beneficial to me as an early childhood educator	4.71	.90	4.77	.64
Future early childhood education students would benefit from this course being integrated into the post-secondary curriculum	4.71	.90	4.78	.66
***Content novelty***				
The course content was new to me	3.77	1.12	3.48	1.14
***Motivation***				
I had a positive attitude toward using the course	4.50	.80	4.74	.51
The course was ***not*** intimidating to use	4.55	.93	4.56	.93
My interest in learning about *physical activity* in early childhood increased as a result of the course	4.65	.84	4.50	.95
My interest in learning about *sedentary behaviour* in early childhood increased as a result of the course	4.52	.89	4.42	.86

*Note. EESS* evaluating e-learning system success, *M* mean, *SD* standard deviation, -- not derived from the EESS model (Al-Fraihat et al., 2020); All items were rated on a 5-point Likert scale (1 = strongly disagree to 5 = strongly agree)

Of the design elements used in the e-Learning course (i.e., text, voiceover, images, animations, videos, within-module knowledge checks, and end-of-module knowledge assessments), most pre-service ECEs communicated that the elements that best facilitated their learning were the images (81.3%) and videos (75.0%), while only 43.8% reported that the animations helped facilitate their learning. In contrast, in-service ECEs communicated that the within-module knowledge checks (81.0%), text (73.6%), and video (73.6%) elements were most supportive to their learning. Like pre-service ECEs, a minority of in-service ECEs (38.0%) reported that the animations facilitated their learning.

### Process evaluation survey implementation outcomes

Across 10 items (ranked on a 5-point Likert scale), pre- and in-service ECEs rated the acceptability of the e-learning course very high on the 5-point scale (*M*_range_ = 4.52 to 4.71 and 4.50 to 4.80 for pre- and in-service ECEs, respectively). Complexity of the course (including its usability, flexibility, clearness of instructions, organization, and conciseness) was also positively rated by both pre-service (*M*_range_ = 4.61 to 4.71) and in-service ECEs (*M*_range_ = 4.47 to 4.79). Pre- and in-service ECEs also demonstrated that they had high self-efficacy to complete the course (*M*_range_ = 4.65 to 4.68 and 4.16 to 4.68 for pre- and in-service ECEs, respectively) and agreed that the course was compatible with their ECE training (*M* = 4.71 [*SD* = .78] and 4.64 [*SD* = .76] for pre- and in-service ECEs, respectively). When asked to rate the perceived effectiveness of the course at facilitating their learning and increasing their physical activity and sedentary behaviour-related knowledge, pre- and in-service ECEs reported high scores (*M*_range_ = 4.42 to 4.73 and 4.45 to 4.74 for pre- and in-service ECEs, respectively). ECEs were also positive about the perceived benefits of the e-Learning course (*M*_range_ = 4.71 to 4.74 and 4.77 to 4.79 for pre- and in-service ECEs, respectively) and reported feeling motivated to both complete the course (*M*_range_ = 4.50 to 4.55 and 4.56 to 4.74 for pre- and in-service ECEs, respectively) and further their learning in physical activity (*M* = 4.65 [*SD* = .84] and 4.50 [*SD* = .95] for pre- and in-service ECEs, respectively) and sedentary behavior (*M* = 4.52 [*SD* = .89] and 4.42 [*SD* = .86] for pre- and in-service ECEs, respectively). Pre- and in-service ECEs provided a moderate rating for the novelty of the course content (*M* = 3.77 [*SD* = 1.12] and 3.48 [*SD* = 1.14] for pre- and in-service ECEs, respectively); however, *SD*s for this item were higher than other items, demonstrating greater variability in participant perspectives. See Table 3 for complete ratings for each implementation determinant/outcome.

### Qualitative perspectives

Twenty distinct themes were referenced by pre- and in-service ECEs (via interviews with 3 and 8 pre- and in-service ECEs, respectively, and text responses in the anonymous survey). These themes represented the following implementation determinants and outcomes: acceptability (*n* = 1 theme), feasibility (*n* = 3 themes), compatibility (*n* = 2 themes), complexity (*n* = 2 themes), context (*n* = 3 themes), perceived effectiveness (*n* = 2 themes), perceived benefits (*n* = 2 themes), motivation (*n* = 2 themes), tension for change (*n* = 2 themes), and relative priority (*n* = 1 theme). Overall, ECEs were very satisfied with the course; one participant noted, “I give it an A++, it was amazing!”, while another commented that “it was the best online workshop I’ve taken.” Further, respondents stated that “the course was straightforward and easy to follow,” while also noting that the e-Learning platform was convenient and “time-friendly” to work into their already busy schedules. However, they also commented on the longer than anticipated duration of the course and suggested that breaking the course into smaller modules would promote motivation and would fit more easily into their schedules. Participants also suggested adding in a discussion forum to make the experience more interactive. While many participants communicated that they appreciated the various design elements (e.g., text, audio, video, external links) in the course, some ECEs reported having technological issues when using a mobile device.

Several ECEs commented on the wealth of new information they learned; one ECE said that they found “lots of topics were new” to them, while another stated that they “did not truly understand the importance of physical activity until [they] took this course.” Even though certain ECEs mentioned that some of the course content was more reinforcement of information they already knew, one ECE noted that it still “gave [them] a new passion for teaching children about physical literacy and the importance of it.” Many ECEs also reported that the course increased their knowledge and confidence to promote physical activity in childcare. For example, one ECE noted that they “love[d] the knowledge it gave [them],” while another commented that “it wasn’t until this course that [they] were actually confident in implementing risky play.” One ECE even mentioned that they have “already started trying to do more active transitions and…active breaks” to reduce prolonged sedentary time in their classroom, highlighting the applicability of the course content to childcare practice. Additionally, many participants stressed the importance of learning this content for those in their profession and that this course would be a welcomed addition to pre-service ECE curricula. For example, one ECE commented that “it should be part of [their] ECE learning right from the college level,” while another reported that the course “could be easily incorporated into an ECE program all across the country.” See Table 4 for example quotations for all themes.

**Table 4:** Pre- and in-service early childhood educators’ perspectives on the implementation of the TEACH study e-learning course

		Example quotes	
Implementation outcome	Theme	Process evaluation survey	Interview
Acceptability	Satisfaction	• “I thoroughly enjoyed all the components of this course. I also thought it was very well put together.” ***(Pre-Service)*** • “I enjoyed the course. I’ve been in the field for 15 years and still found new relevant information in this and that was very exciting.” ***(In-Service)*** • “It was the best online workshop I’ve taken.” ***(In-Service)***	• “I give it an A++, it was amazing!” ***(Pre-Service-1)*** • “Everybody took it, and everybody loved it… including myself. And we were very thankful that we got to do it because it was so interactive, and we learned so much from it.” ***(Pre-Service-2)*** • “Overall, it was all rich and interesting.” ***(In-Service-8)***
Feasibility	Convenience	• “I liked that I was able to work at my own pace. Sometimes I could do one module in one sitting, sometimes I couldn’t, but I appreciate the flexibility.” ***(In-Service)***	• “Anybody who has a computer can do these courses… people can do them kind of in their own time and it's available to more people.” ***(Pre-Service-2)*** • “There's a certain demographic that benefits from having the e-learning opportunity. I mean, if you work full time and if you have, you know—your family life on top of it, taking part in e-Learning courses is much more manageable, you know what I mean? A lot more time-friendly.” ***(In-Service-3)***
	Time commitment	• “It took me longer than 5 hours to complete because of note taking.” ***(In-Service)*** • “Finding time to complete the course during the week was tricky. I work full-time, so it was the weekends when I had the time to complete the course. It seemed to take me longer than the recommended time.” ***(In-Service)***	• “The video library at the end and the resource at the end are all very, very useful. But it did take a little while to get through it all.” ***(In-Service-3)***
	Integration into pre-service ECE programs	• “I would love for this to be a part of students’ learning through their course work while learning/studying to become an early childhood educator.” ***(In-Service)***	• “I think it fits into our courses so well that I think that there could be a whole course that we take over four months and just learn about this. I think it would be very beneficial to educators because even doing this in six, seven hours, my whole outlook kind of changed.” ***(Pre-Service-2)*** • “I think that this could be easily incorporated into an ECE program all across the country.” ***(In-Service-1)***
Compatibility (appropriateness)	Alignment with ECE philosophy	• “Everything was rewarding for our profession.” ***(In-Service)*** • “After being in the early years field actively working with children of various age groups, it was refreshing to know that some of things I have learned haven't changed and I don't have to feel like such a "dinosaur" when I encourage the children to play more instead of them wanting to be glued to their screens all day.” ***(In-Service)***	• “Based on what we're taught in school, it most definitely aligns with our philosophy.” *(* ***In-Service-1)*** • “I think it aligns very well because… everything we do is for the benefit of our children in our care and the families … learning how to maximize their time with us is important. And I think, yeah, it aligns very well with what our philosophies are or should be.” ***(In-Service-3)***
	COVID-19 influence	• “Given the restricting realities facing many children during COVID shutdowns and quarantines, this information is so important and relevant to ECEs right now.” ***(In-Service)***	• “I sit way too much, especially now because of COVID. I'm a hermit crab … I don't leave my apartment ... It really opened my eyes that we shouldn't be sitting as much as we do.” ***(Pre-Service-2)*** • “I think it's very relevant material … especially given the current setting. I mean, we have more and more children who are forced to be sitting at home on their couch now…and I think it's very important for educators and families to be aware of the dangers of not getting your children out and active.” ***(In-Service-3)***
Complexity	Knowledge checks and assessments	• “Some of the end knowledge checks were challenging for first time learners.” ***(In-Service)***	• “They were challenging, which is nice, because … I don't like doing things and just having these knowledge checks that are just like, OK, I know that…I know that…I know that…I know that. It's nice when it's challenging because then you know that you're getting new information.” ***(In-Service-1)*** • “They weren't super easy, but they weren't so hard. So, if you paid attention and focused and did the course and didn't multitask…I thought it was like in the middle.” ***(In-Service-5)***
	E-learning platform	• “The course was really easy to use, which I think is great, especially for people who aren't tech savvy.” ***(In-Service)*** • “The course was straight forward and easy to follow.” ***(In-Service)***	• “I'm…technologically challenged and I got through it quite nicely.” ***(Pre-Service-1)*** • “It's very smooth. Like, yeah…it's very easy to complete it.” ***(Pre-Service-3)*** • “The navigation was very simple. It was easy to follow” ***(In-Service-1)***
Context	E-learning likes	• “This is really awesome! Great presentation, side notes and illustrations that added to visual learning.” ***(In-Service)*** • “I liked the fact that you had to complete a full lesson before moving on. As well as not being able to fast forward was ideal to fully understanding the material.” ***(In-Service)*** • “I really appreciated the additional resource materials-both websites and videos.” ***(In-Service)***	• “The videos were incredible. Like there was a lot of them. And being an online student now, videos are really useful to me, especially because it really hones in the information.” ***(Pre-Service-2)*** • “I liked the external links because those are things you can save for later as well as the audio” ***(In-Service-2)*** • “I think they have a good mixture of text and image and video. So, it's balanced.” ***(In-Service-6)***
	E-learning challenges	• “It was not super compatible with my phone.” ***(In-Service)*** • “There were also a few times where the voiceover couldn't be paused as I was writing things down and I had to begin the whole section again.” ***(In-Service)*** • “Sometimes the audio wouldn't catch up with the slide progression.” ***(In-Service)***	• “I did have a couple issues with the voiceover.” ***(Pre-Service-2)*** • “I feel like the only negative to it is that there's no way to clarify anything and there's no live interaction.” ***(In-Service-5)*** • “If incorrect it didn’t not show the correct answers. So, we had to repeat that.” ***(In-Service-8)***
	Suggestions for improvement	• “It would have been great to have more information on the 0-18month age group.” ***(In-Service)*** • “Break down some content into smaller modules.” ***(In-Service)*** • “More examples from Canadian childcare centres (i.e., videos).” ***(In-Service)*** • “A discussion board section where we can connect with other educators taking the course to further our professional development.” ***(In-Service)***	• "It would be cool to kind of have, like, a PDF resource thing at the end … like a resource of all the different activities that were discussed or something like that.” ***(In-Service-2)*** • “I think it was missing in the e-learning was for the children with the diverse needs. So, the special needs children…like how we can alternate physical activities for them.” ***(In-Service_6)*** • “Maybe you can interview the early educators on what they do to incorporate those skills into the practice—like a testimonial” ***(In-Service-6)***
Perceived effectiveness	Increased confidence	• “I feel more confident in my ability to provide great physical experiences.” ***(In-Service)*** • “Now that I have completed this e-Learning course, and been provided with countless resources, I feel more confident about leading physical literacy interactions in my future endeavors.” ***(Pre-Service)***	• “It wasn't until this course that I was actually confident in implementing risky play.” **(Pre-Service-2)** • “Having a course that's full of strategies and videos and games and examples that show you that really boosted my confidence and being able to do these things with children.” **(Pre-Service-2)** • “I'm more comfortable and confident in my abilities of going outside in [poor weather] and being able to stay engaged in the children's learning.” **(In-Service-7)**
	Increased knowledge	• “I love the knowledge it gave me and the resources for me to expand further as well as ways I can help my families see the importance.” ***(In-Service)*** • “I feel more comfortable with risky play with the knowledge I have taken from this study.” ***(In-Service)*** • “I found lots of topics were new to me. The videos and resource library were very helpful in learning the new concepts.” ***(In-Service)***	• I learned a lot of things that were briefly touched on in my courses, but I learned a lot more in depth.” ***(Pre-Service-2)*** • “…gaining more knowledge on … the guidelines, because it's not necessarily something you talk about in school.” ***(In-Service-2)***
Perceived benefits	Prompted awareness	• “It was all very informative and eye opening.” ***(In-Service)*** • “I liked a lot of resources that provide new ideas for the physical activities. It surprised me sometimes how little effort it might take to get children become physically active.” ***(In-Service)*** • “I did not truly understand the importance of physical activity until I took this course.” ***(In-Service)***	• “A lot of it was reinforcement, but it gave me a new passion for teaching children about physical literacy and the importance of it.” ***(Pre-Service-1)*** • “It really opened my eyes that we shouldn't be sitting as much as we do.” ***(Pre-Service-2)*** • “my co-worker here in preschool and I—we both did this together. And so, we were able to talk about the things we were learning as we were doing it. And we really stood back and watched and were thinking about the different activity levels inside versus outside. And when we stopped and really realized what we were doing and what the kids were doing, we thought—*oh, my gosh, they're right.”* ***(In-Service-3)***
	Useful for training and practice	• “Tons of great info, with tons of resources to be able to go back to in the future.” ***(In-Service)*** • “I enjoyed the amount of links to other sites to get more information on outdoor and risky play and all of the other physical literacy websites – I will be using these!” ***(In-Service)***	• “As an educator… this whole course was great for me … because I learned so much and so many strategies for how to implement this into my everyday work.” ***(Pre-Service-2)*** • “I used a lot of this information in my classes and to help kind of hone in my points and help others when we were doing like a risky play assignment. So even in school, after doing this, of course, I was able to put it into my classwork. And I think being able to do that made me understand it even more because I actually got to use it in something that I was planning.” ***(Pre-Service-2)*** • “I have actually already started trying to do more active transitions and the active breaks. I haven't gathered any of the like the big outdoor loose parts materials, but I have spoken to my administrator about trying to find resources for that because I really enjoyed that part of it” ***(In-Service-1)***
Motivation	Interest in content		• “The [*professional*] learning hours didn’t matter. I just…found the topic interesting.” ***(In-Service-5)***
	Knowledge checks and assessments		• “I wanted to prepare for them even more because I knew there is a test coming up … I wanted to do well, I wanted to ace it.” ***(Pre-Service-1)*** • “I certainly liked, again, the different testing methods to keep you on your toes and make sure you're paying attention” ***(In-Service-3)***
Tension for change	Current issues with practice		• “I would have loved to have learned more about sedentary behavior and physical activity before I started in my career because…like applying it now, yeah it helps the kids I have now. But what about the kids they had before? It didn't help them, right? So, it'd be nice to have it before people go into the work field.” ***(In-Service-4)*** • “The situation is becoming very troubling these days and concerning that, children are spending more time online.” ***(In-Service-8)*** • “You have these superiors over us…who are the ones who decide, not me. A little bit of rain, a few drops or a bit of snow. They would cancel recess just because of that.” ***(In-Service-8)***
	Current issues with pre-service curriculum		• “That's not something that was really touched on in undergrad. So, not a lot of ECEs really know what that is. I think it should be touched on more and this is kind of a great way to segue into that and start the discussion on it.” ***(In-Service-2)*** • “When I went to [*college*] we didn’t do any training…like any physical activity.” ***(In-Service-5)***
Relative priority	Importance of training		• “I think all educators, no matter if you're starting out like me or if you've been in this in the school system for 30 years, I think everybody should take this course because there's so much information and it's so helpful and there's so many strategies for us educators. And I think the more strategies we have as educators, the better educators we become. And it gives the children we work with higher quality care.” ***(Pre-Service-2)*** • “I think that it's a very important topic and it should be learned early in the career.” ***(In-Service-1)*** • “I've already told my supervisor—you really need to do this it's so good! She’s like, really it was that good? I was like, yeah, it was awesome. You should definitely do it.” ***(In-Service-4)***

*Note. ECE* early childhood educator; quotes from the process evaluation survey were submitted anonymously

## Discussion

This process evaluation of the TEACH pilot study aimed to highlight implementation factors that contributed to feasibility of the intervention for scale-up. Both pre- and in-service ECEs exhibited moderate-to-high fidelity to the TEACH study e-Learning course and communicated that the course was highly acceptable, compatible, effective, feasible, and appropriate in complexity. Challenges reported by ECEs included technical difficulties with the e-Learning (LMS) platform when using mobile devices and a longer than anticipated course duration. These results highlight areas of improvement for the e-Learning course and its delivery prior to scale-up in pre-service ECE programs across Canada and offer unique implementation perspectives with respect to online training interventions for ECEs.

Overall, both pre- and in-service ECEs responded well to the e-Learning mode of delivery of the course. They reported that the online training effectively facilitated their learning and made it convenient to work into their schedules. The self-paced nature of the course allowed participants to take notes and review sections of content. The benefits of e-Learning compared to in-person delivery have been echoed in previous online training interventions for ECEs; for example, Kennedy and colleagues [21] and Ward and colleagues [19] both cited that the convenience of online learning supported participation and intervention fidelity among ECEs in their respective studies. Participants in the present study indicated that they thoroughly enjoyed the various design elements and commented that having so many videos and knowledge checks throughout the course supported their learning. However, participants did suggest that adding a discussion forum component to the LMS platform would enhance their experience by making it more interactive, a component of in-person learning they valued. This is consistent with recommendations from Peden et al. [33] which suggested that peer mentoring via forums would promote ongoing discussions and provide a sense of belonging in the ECE community. Therefore, future e-Learning courses for ECEs should consider incorporating such discussion board elements to extend ECEs’ learning beyond what is presented in the course and allow ECEs to network with peers with similar professional learning interests.

In addition to ECEs’ positive perspectives of the e-Learning mode of delivery, the e-Learning course itself showed moderate-to-high fidelity, and dose delivered was close to 100%. These results were encouraging, particularly considering the intervention was delivered during the COVID-19 pandemic, when pre-service ECEs were less engaged in their class community (due to distance learning) and in-service ECEs were tasked with additional responsibilities (e.g., ensuring cleanliness and distancing within their classrooms were maintained). When compared to other online training interventions for ECEs, Hoffman and colleagues [26] reported that 100% of participating ECEs completed their physical activity online training workshop (60 min); however, it is important to note the shorter course duration and that ECEs were able to complete the training during working hours, both of which likely contributed to the high-fidelity reported. In contrast, Kennedy and colleagues [21] reported that for their online training modules, 19 of the 26 participating ECEs (73%) completed the full training, and the average course completion rate (i.e., dose delivered) was 92.6%. The latter findings are more consistent with fidelity and dose delivered results from in-service ECEs in the present study, likely due to the similar course duration and completing the course outside of work hours. Notably, pre-service ECEs in the present study completed the course in fewer days and reported higher intervention fidelity and dose delivered than in-service ECEs—likely a function of being provided class time (in-person or virtually) to complete the course. As such, these findings highlight important considerations, such as time to complete the training, for future implementation in post-secondary ECE programs and as professional learning for in-service ECEs to promote fidelity, feasibility, and dose delivered.

With respect to course content, nearly all topics were reported to be enjoyable by ECEs. However, of note, the large majority of both pre- and in-service ECEs selected both outdoor play and risky play as their favorite topics. This preference is consistent with recent literature, which has echoed the growing interest in outdoor and risky play among those working in early learning settings. For example, Dietze and Kashin [34] analyzed discussion forum responses from Canadian ECEs (*n* = 207) who participated in an online course in outdoor play pedagogy; participants communicated that formal training in outdoor play was lacking from their post-secondary program and that participating in the online course gave them new knowledge in this area. ECEs in Dietze and Kashin’s study [34] also agreed that those in their profession should be made more aware of the importance of outdoor and risky play in early childhood, noting the importance of overcoming hesitancies of risk-averse colleagues and parents through education. These findings are similar to those from the present study, where ECEs suggested that taking the TEACH study e-Learning course increased their comfort levels with risky play, while they also recommended that all ECEs should take the course. As such, increased opportunities for outdoor and risky play-related education, via formal pre-service schooling and professional learning opportunities, seem to be desired by ECEs to build their capacity to support these types of active play experiences for children in their care.

In addition to ECEs’ reported interest in the course content, both pre- and in-service ECEs communicated that this type of education is important and necessary for all ECEs. Yet, many participants voiced their concerns over not having learned much about physical activity or sedentary behavior during their pre-service schooling. Participants noted that topics relating to physical activity and sedentary behavior were often mentioned, but not discussed in any substantive detail. These perspectives confirm the findings from Bruijns et al. [35] who found that only 32.2% and 26.7% of Canadian pre-service ECEs (*n* = 1292) reported having received physical activity and screen-viewing-related education in their college/university ECE program, respectively. Consequently, in-service ECEs have consistently requested to receive additional training and support in these areas [34, 36, 37]. However, it was encouraging to find that many TEACH pilot study participants were optimistic about the feasibility of integrating this e-Learning course into pre-service ECE programs and that the course aligned well with ECE philosophy. While a number of childcare-based interventions have used professional development to enhance intervention effectiveness [38], ensuring ECEs receive comprehensive education about physical activity and sedentary behavior in their formal schooling is important to help scaffold their development of a health-promoting teaching philosophy.

### Strengths and limitations

While this pilot study has many strengths, such as the inclusion of both pre- and in-service ECEs and the evaluation of 13 distinct implementation outcomes and determinants via triangulation of e-Learning metrics, survey, and interview data, this work’s limitations must be discussed. First, this study was conducted during the second and third waves of the COVID-19 pandemic in Canada, when post-secondary ECE programs were mainly delivered virtually and in-service ECEs were tasked with additional responsibilities at their workplaces. As such, pre-service ECEs were not as engaged with their program instructors (who helped facilitate students’ recruitment and participation), resulting in a lower than anticipated sample size. Further, due to the increased workplace demands, in-service ECEs lacked time to be able to complete the course in the recommended timeframe, resulting in lower course completion rates (i.e., fidelity) and longer course completion timeframes (i.e., feasibility). Second, the small pre-service ECE sample size limited the number of volunteers that could be invited to participate in an interview. Due to competing demands of schoolwork and family commitments, only three participants volunteered; therefore, saturation in this study population could not be reached. Third, volunteer bias may have been present for the interview data, as it is more likely that participants who had a positive experience with the course volunteered to discuss their experiences with it than those who may have had a more negative experience. Fourth, it is possible that recruitment methods (i.e., social media and email advertisements) for in-service ECEs may have introduced selection bias, as this may have unduly targeted in-service ECEs already familiar with online platforms. Finally, while a diverse sample of both pre- and in-service ECEs was achieved, results from this study may not be generalizable to a future full-scale study sample or other research with this population.

### Research implications and future directions

The TEACH e-Learning course may be the first online professional learning opportunity that covers a broad range of movement behavior concepts in early childhood, including, but not limited to, physical activity, sedentary behavior, 24-h movement behavior guidelines, physical literacy, fundamental movement skills, outdoor play, risky play, and loose parts play. As such, there is great potential for this course to be adapted for use in other countries, particularly in countries where 24-h movement guidelines have been adopted. As the objectives of this pilot study were to improve broader implementation by gathering feedback about the e-Learning course content, delivery, and select implementation elements during a small window of time, reach, adoption, and sustainability of the e-Learning course could not be explored. However, with 48 pre-service and 125 in-service ECEs having completed the course, over 1000 young Canadian children (based on Ontario’s ECE to preschooler ratio of 1:8 [39]) will have ECEs who are more knowledgeable and confident in facilitating active opportunities in the childcare setting. Longer-term implementation of the e-Learning course and assessing changes to childcare practices of participating ECEs will be key to determining whether the TEACH e-Learning course is a sustainable and effective professional learning initiative. Further, implementing in a larger sample of pre-service ECE programs, and including perspectives of ECE program instructors, will help determine the feasibility and appropriateness of integrating the TEACH e-Learning course into post-secondary ECE curricula across Canada.

## Conclusion

In conclusion, the TEACH e-Learning course appeared to be an implementation success and pre- and in-service ECEs were highly satisfied with their experience. Despite some technical difficulties experienced by a small number of learners, participants reported that the course effectively facilitated their learning, was appropriate in complexity and presented content that was both interesting and important for their professional development. Additionally, participants enjoyed that the e-Learning course had many interactive elements and that it was convenient for them to work into their schedules. These findings demonstrate the value of e-Learning for ECEs’ professional development. Participant suggestions and perspectives of the TEACH e-Learning course will be used to make improvements prior to future implementation with larger sample of pre- and in-service ECEs. Given the overwhelmingly positive feedback from participants, it is clear that Canadian ECEs are in need of more professional learning and development opportunities in physical activity and sedentary behavior. As such, implementation and scale-up determinants and outcomes will need to be top of mind when expanding this training to promote reach, adoption, and sustainability of the TEACH e-Learning course.

## Supplementary Information

**Additional file 1.**

## Data Availability

The datasets generated and/or analyzed during this current study are not publicly available due to ethical restrictions but are available from the corresponding author on reasonable request.

## Abbreviations

CFIR: Consolidated Framework for Implementation Research
ECE: Early childhood educator
LMS: Learning management system
MVPA: Moderate-to-vigorous physical activity
TEACH: Training pre-service EArly CHildhood educators in physical activity

## Footnotes

1 Does not match recruitment sample due to some participants selecting the wrong ECE level during sign-up
